# An Adolescent Sensitive Period for Social Dominance Hierarchy Plasticity Is Regulated by Cortical Plasticity Modulators in Mice

**DOI:** 10.3389/fncir.2021.676308

**Published:** 2021-05-12

**Authors:** Lucy K. Bicks, Michelle Peng, Alana Taub, Schahram Akbarian, Hirofumi Morishita

**Affiliations:** ^1^Department of Psychiatry, Icahn School of Medicine at Mount Sinai, New York, NY, United States; ^2^Department of Neuroscience, Icahn School of Medicine at Mount Sinai, New York, NY, United States; ^3^Department of Ophthalmology, Icahn School of Medicine at Mount Sinai, New York, NY, United States; ^4^Mindich Child Health and Development Institute, Icahn School of Medicine at Mount Sinai, New York, NY, United States; ^5^Friedman Brain Institute, Icahn School of Medicine at Mount Sinai, New York, NY, United States

**Keywords:** social hierarchy, plasticity, development, prefrontal cortex, thalamus

## Abstract

Social dominance hierarchies are a common adaptation to group living and exist across a broad range of the animal kingdom. Social dominance is known to rely on the prefrontal cortex (PFC), a brain region that shows a protracted developmental trajectory in mice. However, it is unknown to what extent the social dominance hierarchy is plastic across postnatal development and how it is regulated. Here we identified a sensitive period for experience-dependent social dominance plasticity in adolescent male mice, which is regulated by mechanisms that affect cortical plasticity. We show that social dominance hierarchies in male mice are already formed at weaning and are highly stable into adulthood. However, one experience of forced losing significantly reduces social dominance during the adolescent period but not in adulthood, suggesting adolescence as a sensitive period for experience-dependent social dominance plasticity. Notably, robust adolescent plasticity can be prolonged into adulthood by genetic deletion of *Lynx1*, a molecular brake that normally limits cortical plasticity through modulation of cortical nicotinic signaling. This plasticity is associated with increased activation of established nodes of the social dominance network including dorsal medial PFC and medial dorsal thalamus evidenced by increased c-Fos. Pharmacologically mediated elevation of cortical plasticity by valproic acid rapidly destabilizes the hierarchy of adult wildtype animals. These findings provide insight into mechanisms through which increased behavioral plasticity may be achieved to improve therapeutic recovery from psychiatric disorders that are associated with social deficits.

## Introduction

Adult mice living in social groups form naturally occurring dominance hierarchies which are highly stable across time (Lindzey et al., 1961; Wang et al., 2011; Williamson et al., 2016; Zhou et al., 2018). Hierarchy formation and maintenance is a complex behavior that relies on recognition of dominance relationships of others and behavioral plasticity in response (Wang et al., 2014; Bicks et al., 2015; Zhou et al., 2018). Previous research has demonstrated that dominance rank is dependent on prefrontal cortex (PFC) circuitry acting in conjunction with midline thalamic structures (Wang et al., 2011; Zhou et al., 2017; Kingsbury et al., 2019; Nelson et al., 2019). However, very little is known about the mechanisms that establish and maintain the stability of these hierarchies. While dominance is a behavioral trait of an individual, the stability of the hierarchy is a property of the group as a whole and is dependent on the change in dominance status of all the individuals within the group. Here we set out to examine the neural mechanisms in control of hierarchy stability as a model for social behavior plasticity in mouse.

While much research in primary sensory cortical areas has outlined the specific mechanisms for regulating experience-dependent development and plasticity of circuitry mediating sensory processing (Hensch, 2004; Morishita and Hensch, 2008), mechanisms regulating experience-dependent development of PFC circuits are still poorly understood. Social experience in the post-weaning juvenile and adolescent periods has profound effects on the development of adult PFC circuitry and social behaviors (Makinodan et al., 2012; Bicks et al., 2020; Morishita, 2020; Yamamuro et al., 2020), suggesting that the circuitry regulating hierarchies may be sensitive to social experience during this period. Whether hierarchies are more plastic during development, or whether modulators of cortical plasticity impact hierarchy plasticity is as of yet unknown.

Several molecules have been identified as cortical plasticity modulators in primary sensory cortical areas, including several molecular “brakes” which actively limit plasticity as the critical period closes (Bavelier et al., 2010). One such plasticity brake is Lynx1, a nicotinic modulator that facilitates closure of the visual critical period (Morishita et al., 2010). Lynx1 knockout (KO) mice, have an open-ended critical period and therefore provide an opportunity to study the effects of elevated cortical plasticity in the adult (Morishita et al., 2010; Bukhari et al., 2015; Sajo et al., 2016; Takesian et al., 2018). Pharmacological interventions, such as Valproic Acid treatment, can reopen critical period cortical plasticity in adults, allowing for interrogation of the impacts of high cortical plasticity on adult behavior (Putignano et al., 2007; Silingardi et al., 2010; Yang et al., 2012; Gervain et al., 2013). For example, in humans, administration of VPA reopens a critical period for auditory learning, allowing for acquisition of absolute pitch in adults (Gervain et al., 2013). It is currently unknown whether critical periods in association cortical areas (including the PFC) are regulated by mechanisms that are shared or distinct to those that regulate primary sensory critical periods. Understanding the mechanisms that regulate plasticity of the PFC and behaviors regulated by the PFC could provide invaluable tools to leverage plasticity mechanisms to improve cognitive and social functioning in adults.

Here we examine the development of mouse social dominance hierarchies and their regulation by critical period modulators, Lynx1, and VPA. We show that baseline mouse hierarchies are stable at an early developmental stage, but show experience-dependent plasticity in response to an experience of losing during the adolescent window. Experience-dependent plasticity is dampened in the adult animal but can be extended into adulthood by genetic absence of Lynx1, while unmasking plasticity in adulthood using the pharmacological treatment VPA leads to destabilization of the baseline hierarchy in mice. Our findings show the first evidence that modulators of sensory critical period plasticity also regulate social behavior plasticity.

## Materials and Methods

### Animals

Juvenile and adult (14 days to 5 months after birth) WT (C57Bl6, Charles River, Wilmington, MA, United States) and adult Lynx1KO (gifted by Dr. Nathaniel Heintz: Rockefeller University) male mice were maintained on a 12-h light/dark (LD) cycle and had access to food and water *ad libitum*. Mice were group housed, 4 in a cage, for at least two weeks after shipping prior to any behavioral experimentation. Information on original litter size was not available. All experiments were approved by the Icahn School of Medicine at Mount Sinai Ethical Committee for animal research.

### Behavior

Mice were first habituated to the tube and the testing environment by placing the subject at one end of the tube and allowing each mouse to run the length of the tube. The direction was alternated between turns. Habituation was completed after successful completion of the task four times. Each day of testing involved pairwise assessment of all possible matches in the tube-test (six total matches). The order of the matches was determined randomly and varied each testing session. Testing began following one training session. Each match was started when both mice fully entered the tube on opposite sides, and ended when one mouse had forced the other to retreat with all four paws out of the plastic tube. Mice were tested in a Plexiglas tube measuring 1 ft in length. The diameter was adjusted depending on the developmental age (0.75 – 1.25 inches internal diameter, Interstate Plastics). Following every trial, the apparatus was cleaned with a cleaning MB-10 solution (Virkon S, Dupont, Wilmington, DE, United States). Developmental tests were conducted weekly, while Valproic Acid (VPA) and Lynx1KO experiments were conducted every other day. To assess experience-dependent hierarchy plasticity, baseline hierarchies were established based on every other day testing for three days, and rank was determined based on number of wins on the final day. The following day, the top ranked animal was forced to lose to the third ranking animal by placing a stopper at the end of the tube, such that the top rank animal would be forced to back out. We assessed hierarchies the following day by again testing pairwise dominance between all pairs in the tube-test.

### Valproic Acid Administration

Valproic Acid (VPA), (200 mg/kg, dissolved in sterile saline) or Saline was injected i.p. every 12 h for 8 days.

### Statistical Analyses

David’s scores (DSs) were assessed using the EloRating R package (Gammell et al., 2003). Generalized linear models were used to assess effects of treatment using the “glmer” function of the lme4 R package. Generalized linear models included the following random effects: animal nested within cage and test date. Generalized linear models including treatment (genotype or drug treatment) as an effect were compared to reduced models not including treatment. Differences between Pearson’s correlations were assessed using the cocor R package to assess Pearson and Filon’s z.

### Immunohistochemistry

Mice were deeply anesthetized with isoflurane and perfused transcardially with 0.1M phosphate-buffer saline (PBS, pH = 7.4) followed by 4% paraformaldehyde in PBS. Brains were removed and transferred to the same fixative. 4–6 h later they were cryoprotected in 30% sucrose in PBS.

All rank one mice were analyzed from 7 cages. Coronal brain sections (35μm) were collected in serial sections using a 24 well plate using a cryostat (Leica). Samples were washed two times with TBS for 10 min and then blocked with (1% BSA/0.25% triton-X 100/TBS) for 1 h at RT. Sections were then exposed to primary antibody (rabbit anti-c-Fos antibody 1:5000) in.1% triton-X 100/TBS at 4°C on a rotator for 20 h. Sections were then washed with a blocking buffer for 10 min, three times at RT. Next, sections were exposed to the secondary antibody (donkey anti-rabbit IgG1:400) (Life Technologies, A21206, Lot#1182675) in.25% triton-X/TBS at RT for 2 h. Slices were washed in TBS for 10 min two more times. Sections were transferred onto glass slides, air dried, mounted with Fluoromont-G Dapi (Southern Biotech) and coverslipped.

### Imaging and Quantification

Images were acquired on a LSM780 Microscope using Zen 2012 software. Tilescans of mPFC slices were captured and Images were analyzed using Fiji. Using the DAPI channel, brain regions of the mPFC were drawn. Bregma range [−0.08 to 3.45] was included in the calculation. Dorsal Anterior Cingulate (dACC) and the Mediodorsal nucleus of the thalamus (MD) were drawn according to the Allen Brain Atlas. The cFos channel was selected: the image was converted to an 8bit type, noise was removed and background was subtracted. Each region was individually selected and the number of nuclei counts was recorded. Imaging and quantification was performed by a blinded experimenter.

## Results

### Experience-Dependent Plasticity of Social Dominance Hierarchy in Adolescent Mice

**FIGURE 1:**
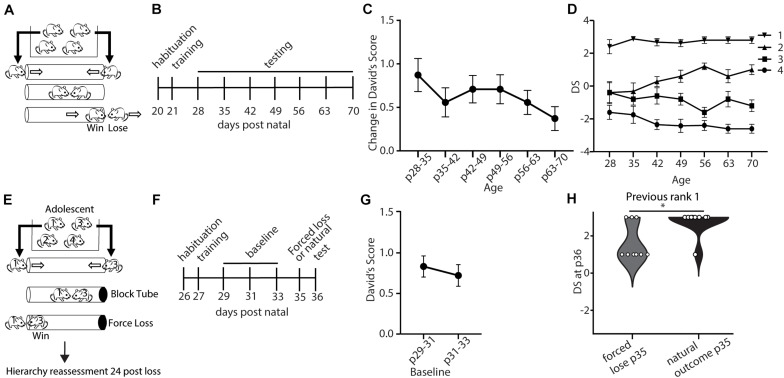
Dominance shows developmentally regulated experience-dependent plasticity. **(A)** Mice within a cage were assessed in the tube test using a round-robin design to assess dominance between each pair in a cage. **(B)** Timeline showing habituation to the tube and testing environment and training, followed by weekly tests of hierarchy across development. **(C)** Weekly changes in average David’s Score (DSs) across development (generalized linear model, *p* = 0.739, *n* = 43 mice in 11 cages) **(D)** DS for each rank assigned based on the average DSs across the testing period. **(E)** Experience-dependent changes in hierarchy for adolescent mice were assessed by altering the outcome of the rank 1 vs. 3 match-up by blocking the tube, forcing the rank 1 animals to lose. Hierarchies were reassessed 24 h later (*n* = 72 mice in 18 cages). **(F)** Timeline showing habituation to the tube and testing environment, training, three baseline tests of hierarchy every other day, followed by forced loss manipulation or natural outcome and dominance status reassessment. **(G)** Baseline hierarchies showed no significant differences in change in David’s Score between p29–31 and p31–33 (generalized linear model, *p* = 0.614, *n* = 72 mice from 18 cages). **(H)** Adolescent rank 1 mice that experienced forced loss showed significantly lower DSs compared with mice that experienced the natural outcome (Wilcoxon signed rank test, ^∗^*p* = 0.02, *n* = 9 mice from 18 cages).

We first set out to evaluate the baseline stability of social hierarchy in mice from the juvenile period following weaning to adulthood. We assessed dominance between all pairs of male mice within a cage of 4 mice in the tube-test (Lindzey et al., 1961) (Figure 1A). Dominance scores derived from behavior in the tube test have been previously shown to correlate well with measures of dominance produced by other assays such as scent marking in the presence of a female and competition for a warm spot (Wang et al., 2011; Wang et al., 2014). A social dominance score for each mouse was quantified by calculating DSs (David, 1987) which are based on the number of wins, and the relative numbers of wins and losses of the opponent. With 4 mice in a cage, the DS ranges from −3 to 3, with a score of −3 representing a mouse who lost all matches and a score of three given to a mouse that wins all matches. Tube tests were repeated weekly following weaning through adulthood (Figure 1B). To assess the stability of social hierarchy across development, we first analyzed the weekly change in DSs (Figure 1C). We observed a modest, but not significant, decrease in the weekly change in DSs across development (Figure 1C). Even in the juvenile period, DSs were stable, with an average change of less than one, which is less than a single rank change in a cage of four mice. For example, in a cage with no ties, a rank 3 to rank 4 change is equivalent to a change in DS from −1 to −3. We further examined the specific ranks (based on the average DS throughout the testing period) and saw that the rank 1 in particular was highly invariable (Figure 1D). This finding demonstrates the presence of stable hierarchies already formed during development (Figures 1C,D). Critical period plasticity is often not observed under baseline conditions, and is characterized by experience-dependent changes (Gordon and Stryker, 1996). Therefore, we tested if a forced experience of losing could disrupt the rank of adolescent mice. After determining baseline rank in each cage, we manipulated the outcome of the tube-test trial by blocking one end of the tube such that the rank 1 animal would need to back out and the lower rank animal (rank 3) would need to walk out, creating an experience of “loss” for the rank 1 and an experience of “win” for the rank 3 animal, and we compared this to an unmanipulated test (“natural outcome”) (Figures 1E–G). We found that adolescent mice (p35) showed significantly reduced DSs one day following forced loss, compared with the natural loss controls (Figure 1H). Overall, these findings suggest that stable social hierarchies are formed early in the juvenile period, but that these hierarchies are plastic to experience during the adolescent period.

### Prolonged Experience-Dependent Plasticity of Social Dominance Hierarchy Into Adulthood by Genetic Deletion of Lynx1, A Cortical Plasticity Regulator

**FIGURE 2:**
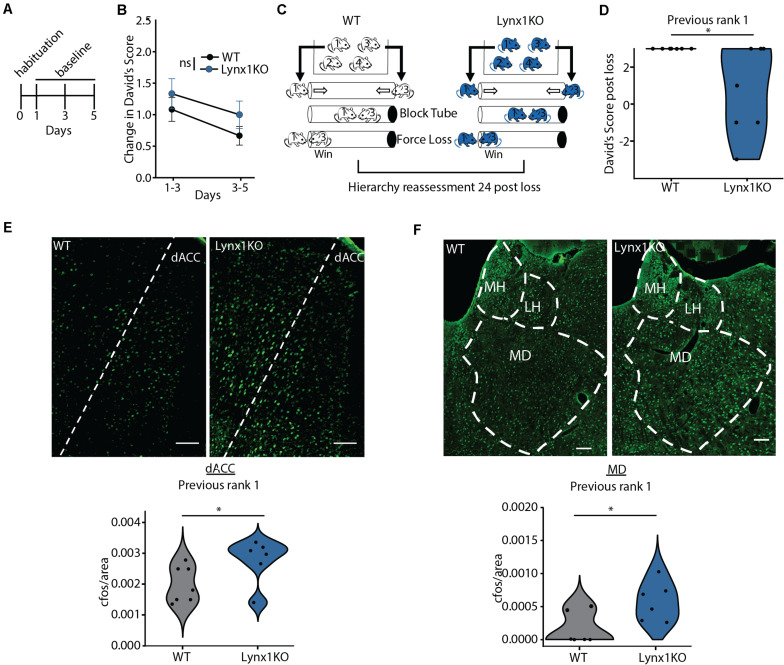
Cortical plasticity modulator Lynx1 limits experience-dependent plasticity of social hierarchy in adult mice. **(A)** Adult WT or Lynx1KO mice were tested in a round-robin design to assess dominance between all pairs in a cage. Mice were habituated to the tube and then baseline hierarchy was assessed every other day for 5 days. Baseline day 1 was training. **(B)** Baseline hierarchies showed no significant differences in change in David’s Score (DS) between genotypes (generalized linear model, *p* = 0.323, *n* = 36 Lynx1KO from 9 cages, *n* = 48 WT from 12 cages). **(C)** Experience-dependent changes in hierarchy were assessed by altering the outcome of the rank 1 vs. 3 match-up by blocking the tube, forcing the rank 1 animals to lose. Hierarchies were reassessed 24 h later. **(D)** WT previous rank 1 mice maintained their rank following loss while Lynx1KO mice showed significantly reduced DS 24 h post loss (Wilcoxon signed rank test, ^∗^*p* = 0.03, *n* = 7 WT mice, 7 Lynx1KO mice). **(E)** Dorsal Anterior Cingulate (dACC) shows significantly increased c-Fos labeling in adult Lynx1KO mice following forced loss (two-tailed un-paired student’s *t*-test, ^∗^*p* = 0.05, *n* = 7 WT mice, *n* = 6 Lynx1KO mice). **(F)** Mediodorsal thalamus (MD) shows significantly increased c-Fos in adult Lynx1KO mice following forced loss (two-tailed unpaired student’s *t*-test, ^∗^*p* = 0.02, *n* = 7 WT mice, *n* = 6 Lynx1KO mice).

We next aimed to determine the mechanism supporting adolescent experience-dependent dominance plasticity. Given that hierarchies show experience-dependent changes during adolescence (Figure 1H), we hypothesized that hierarchy stability may be regulated by modulators of cortical plasticity, which are typically high during juvenile windows and decrease in adulthood. To test this, we leveraged a model of open-ended critical period cortical plasticity, the Lynx1KO mouse. Lynx1 is a nicotinic modulator that increases during adolescence, providing a brake on cortical plasticity in adults. Adult Lynx1KO mice have an open-ended critical period for cortical plasticity, allowing for juvenile-like plasticity in primary sensory cortical areas in adult animals (Morishita et al., 2010; Bukhari et al., 2015; Sajo et al., 2016; Takesian et al., 2018). To test whether social hierarchies may be governed by shared neurobiology underlying critical period cortical plasticity in primary sensory areas, we first assessed baseline hierarchy stability in adult WT and Lynx1KO mice following habituation and training (Figure 2A). There were no significant differences in baseline change in DSs across groups, mirroring adolescent data (Figure 2B). Next, we employed our “forced-lose” assay to assess experience-dependent changes in dominance in Lynx1KO and WT adult male mice (Figure 2C). We then re-tested the hierarchy 24 h later and found that compared to adult WT rank 1 mice, adult Lynx1KO rank 1 mice showed significantly reduced DS 24 h after unexpected experiential loss (Figure 2F). These findings demonstrate significant plasticity in social hierarchy based on experience in adult animals with open-ended critical period for cortical plasticity, suggesting modulators of cortical plasticity may regulate dominance hierarchy stability.

### Brain Regions Involved in Control of Social Dominance Show Differential Response to Loss in a Model of Open-Ended Cortical Plasticity

Next, we aimed to determine the brain regions associated with the plasticity of social hierarchy regulated by Lynx1. Convergent evidence shows regions of the dorsal medial PFC, including the dorsal Anterior Cingulate Cortex (dACC), acting in concert with the medial dorsal thalamus (MD) are essential for regulating rank within a hierarchy (Wang et al., 2011; Zhou et al., 2017; Nelson et al., 2019). We therefore tested whether these regions show differential activation after experiencing an unexpected loss in the tube test between WT animals, who go on to maintain their rank, and Lynx1KO animals, whose rank decreases in the following 24 h (Figure 2D). We perfused Lynx1KO and WT rank 1 animals that had experienced a forced loss 90 min prior in order to assess early immediate gene c-Fos activation in the dominance network, including the dACC and MD (Figures 2E,F). We found that Lynx1KO animals had increased c-Fos activation in both the dACC and the MD, suggesting activation of the dominance network may be important for encoding the experience of loss leading to a subsequent decrease in dominance (Figures 2E,F). Overall, our findings suggest that experience-dependent plasticity of social hierarchy in adult mice is limited by the cortical plasticity modulator Lynx1 and is associated with corticothalamic network activity.

### A Pharmacological Modulator of Cortical Plasticity, Valproic Acid, Induces Plasticity in Adult Social Hierarchy

**FIGURE 3:**
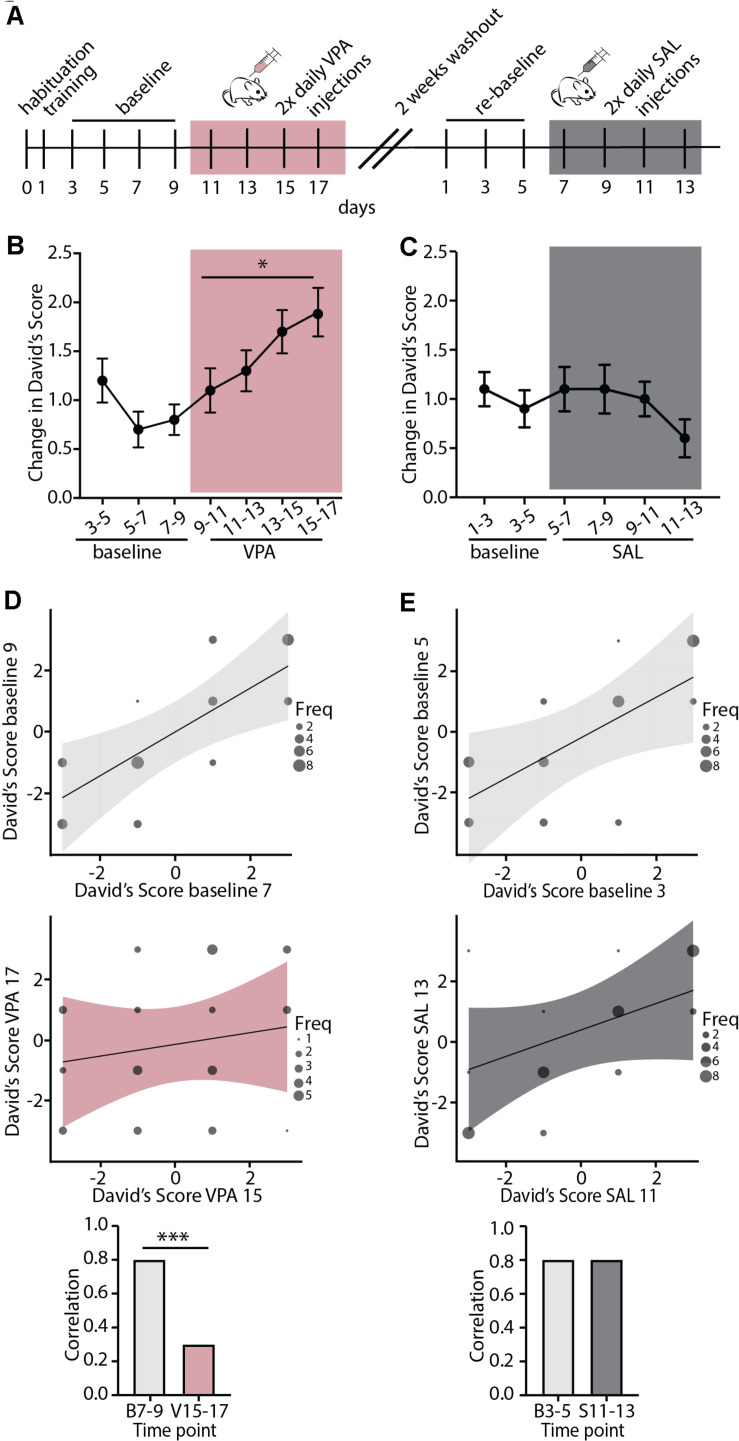
Valproic Acid, a pharmacological modulator of cortical plasticity, destabilizes hierarchy stability. **(A)** Timeline showing habituation and training, followed by baseline testing and testing under Valproic Acid (VPA) 2x daily injection followed by 2 weeks of drug wash-out. Mice were then re-tested for a baseline period and a saline (SAL) 2x daily injection control treatment. **(B)** VPA treatment increased change in DSs separated by a day, an indication of increased hierarchy instability while **(C)** injection with SAL had no effect on hierarchy stability (VPA: generalized linear model, ^∗^*p* = 0.02 SAL: generalized linear model, *p* = 0.68, *n* = 40 mice in 10 cages). **(D,E)** Baseline testing sessions separated by a day show highly correlated DS values (**D,E,** top) while post-VPA treatment DS correlations dissolve (**D,** middle). SAL treated mice retain correlated DSs (**E,** middle). Baseline DSs are significantly more correlated than DSs following VPA treatment (**D**, bottom, Pearson and Filon’s z, ^∗∗∗^*p* = 0.0005) while SAL treatment does not affect DS correlations (**E**, bottom, *p* = 0.5374).

We next set out to test whether regulation of the stability of mouse dominance hierarchies is broadly regulated by shared neurobiology in control of cortical plasticity, or is specifically regulated by nicotinic acetylcholine modulation by Lynx1. To this end, we injected WT mice with Valproic Acid (VPA), a pharmacological manipulation that is known to re-open adult visual and auditory critical periods (Putignano et al., 2007; Silingardi et al., 2010; Yang et al., 2012; Gervain et al., 2013). Mice were injected twice daily for 8 consecutive days and underwent the tube test every other day (Figure 3A). We found that VPA treatment significantly increased the change in DS, indicating destabilization of the baseline hierarchy (Figure 3B). We then allowed all cages to re-stabilize their hierarchies with a 2-week washout period with no handling, re-established baseline DSs, and began saline injections twice daily for eight consecutive days (Figure 3A). Saline injection did not destabilize hierarchies, showing a specific effect of VPA on hierarchy stability. DSs were highly correlated in both baseline periods (Figures 3D,E **top panels)**, but following VPA treatment, correlations across tests began to breakdown, showing uncorrelated hierarchies after one week of VPA treatment, but not following Saline treatment (Figures 3D,E **middle panel**). This led to a significant difference between strength of correlation at baseline and following VPA treatment, but not following Saline treatment (Figures 3D,E **bottom panel**). Therefore, adult re-opening of juvenile-like cortical plasticity with VPA treatment destabilizes hierarchies without further manipulation (such as unexpected experience of losing which was necessary to induce plasticity in hierarchy in adult Lynx1KO mice and adolescent WT mice). This difference may reflect the timing and the nature of manipulations; while Lynx1KO results in open-ended cortical plasticity, VPA administration re-open plasticity in the adult after the critical period had closed. Alternatively, the difference in findings between Lynx1KO and VPA administration could be due to the nature of the manipulation: While Lynx1KO removes one of many brakes on cortical plasticity (Bavelier et al., 2010), VPA administration is a broad manipulation that has multiple targets. Overall, our findings support the model that the stability of mouse social dominance hierarchies is broadly regulated by the neurobiological mechanisms that control cortical plasticity.

## Discussion

**FIGURE 4:**
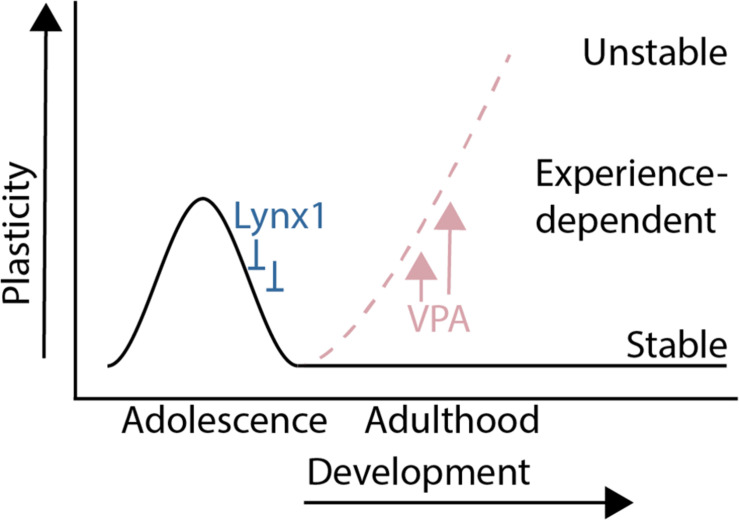
Summary: A sensitive period for adolescent social dominance plasticity is regulated by cortical plasticity modulators.

The results from this study show that a sensitive period for experience-dependent social dominance plasticity of male mouse social dominance hierarchies is regulated by molecular modulators of cortical plasticity that are known to regulate critical periods in sensory cortex (Figure 4). Lynx1, a developmentally regulated brake on critical period plasticity, regulates experience-dependent change in social dominance rank, as hierarchies of Lynx1KO mice are destabilized by a single experience of forced-losing while WT hierarchies remain intact. Forced losing activates regions known to be involved in dominance such as the dmPFC and midline thalamic structures. Pharmacological re-opening of critical period cortical plasticity rapidly destabilizes hierarchy structures at baseline, showing that, in the adult animal, decreased plasticity helps to maintain a stable dominance relationship within the cage. Our findings provide evidence of cortical plasticity modulators directly altering behavior in a social context and shed light on our understanding of the developmental tradeoff between behavioral stability and plasticity in the face of changing contexts.

Previous studies on mouse social dominance hierarchies have studied ways in which rank can be changed by manipulating molecular and circuit mechanisms (Timmer and Sandi, 2010; Wang et al., 2014; Yamaguchi et al., 2017a,b; Zhou et al., 2018). For example, rank can be increased by increasing AMPA receptor mediated synaptic transmission within the dmPFC (Wang et al., 2011), or by manipulating the MD to dmPFC projection (Zhou et al., 2017; Zhou et al., 2018; Nelson et al., 2019). These studies have demonstrated that a dominant animal can lose its’ status, or a subordinate animal can gain rank. In our study, instead of focusing on the dominance rank of individual animals *per se*, we studied the rank stability within a group hierarchy. Unlike manipulations previously discussed, adult manipulations of cortical plasticity in this study change the overall stability of the hierarchy, leading to high amounts of rank change regardless of the direction of change. This study therefore adds to a growing body of knowledge about the mechanisms mediating male mouse hierarchies by providing evidence that increased cortical plasticity leads to hierarchy destabilization. While this study manipulated cortical plasticity broadly, we observed convergence onto known circuits that mediate dominance rank, suggesting increased activation of these circuits is associated with higher plasticity of the hierarchy within the cage.

Our results show that a genetic brake on plasticity, Lynx1, can be removed leading to high experience-dependent dominance plasticity in adult male mice. As Lynx1 is a modulator of nicotinic signaling in the cortex and desensitizes cells to acetylcholine responses (Miwa et al., 1999; Ibañez-Tallon et al., 2002; Miwa et al., 2006), our findings suggest a key role of nicotinic signaling in regulating hierarchy plasticity. Previous studies have shown that nicotinic acetylcholine receptors (nAChRs), which are the target of Lynx1 binding, tend to localize on cortical neurons and presynaptic thalamocortical terminals (Ibañez-Tallon et al., 2002; Disney et al., 2007). Consistently, our c-Fos studies in Lynx1KO mice showed that the experience of losing elicited higher c-Fos activation in the dACC and the MD compared with WT animals, brain regions that were previously shown to respond to previous wins to update dominance status (Zhou et al., 2017). Other studies that manipulated the social context to induce hierarchy plasticity have also shown activity of the PFC during times of changing social rank, confirming the PFC is a key region involved in updating dominance rank in response to social experience (Williamson et al., 2019). Together, our study suggests that a plasticity brake, known to regulate sensory cortex plasticity, can gate experience-dependent plasticity in the PFC circuit known to regulate dominance status. It should be noted that our study using c-Fos staining has limited temporal and spatial resolution. Future studies are necessary to examine to what extent c-Fos activation following forced lose accompanies changes in synaptic efficacy as well as network level oscillations between dACC and MD by incorporating electrophysiological methodology.

Our study also applied a pharmacological approach (VPA administration) to support a converging role of critical period plasticity regulators in inducing plasticity in social dominance hierarchy. While both genetic (Lynx1KO) and pharmacological (VPA) manipulations induced higher instability of mouse hierarchies, there were distinct differences between the outcomes of the two manipulations: Unlike Lynx1 loss, pharmacologically mediated reopening of the critical period through VPA treatment destabilizes baseline hierarchy instability (Figure 3). This difference could be due to the developmental timing of heightened cortical plasticity between these two models: Since Lynx1KO animals lack a break which normally facilitates the closure of the critical period, these animals show “open-ended” cortical plasticity while VPA treated adult WT animals would show a normal critical period closure, followed by an adult reopening of critical period plasticity. This developmental difference could lead Lynx1KO animals to compensate for elevated cortical plasticity across development through, for example, other known plasticity brakes such as PirB and NgR (McGee et al., 2005; Syken et al., 2006; Bavelier et al., 2010). Future studies are warranted to examine the contribution of these plasticity brakes to social dominance hierarchy plasticity. Adult increases in cortical plasticity following VPA treatment may sensitize animals to social experiences in the cage, leading to frequent updating of dominance relationships and high baseline hierarchy instability. Over time VPA-treated animals may show a stabilization of the baseline hierarchy and a similar pattern to Lynx1KO animals in which baseline hierarchies are stable and a manipulation of social experience is needed to unmask increased plasticity. Alternatively, VPA treatment may be a more potent destabilizer of hierarchy plasticity due to its mechanism of action and not the developmental timing of the manipulation. VPA has multiple mechanisms of action including enhancement of inhibition in the cortex and inhibition of histone deacetylases which can broadly alter the epigenome and lead to increased transcriptional plasticity (Phiel et al., 2001; Machado-Vieira et al., 2011). Whether through direct action on GABAergic signaling or through transcriptional responses due to increased acetylation, VPA is known to regulate oscillation activity in frontal cortex (Béla et al., 2007). Future studies are needed to assess whether GABAergic facilitation or HDAC inhibition or both simultaneously are required to disrupt hierarchy stability in adult mice. VPA is an anti-epileptic drug as well as an approved mood stabilizer and has been used as a treatment for Bipolar disorder (Phiel et al., 2001; Chiu et al., 2013). Future studies are warranted to determine if VPA treatment would provide an effective treatment to treat social deficits. It is also important to investigate the contribution of other pharmacological interventions known to reactivate sensory cortical plasticity in adulthood (e.g., selective serotonergic reuptake inhibitors, chondroitinase) (Pizzorusso et al., 2002; Maya Vetencourt et al., 2008) to social dominance hierarchy plasticity.

One of the limitations of this study is that we did not test if social experience impacts the opening or closure of sensitive period for social dominance hierarchy plasticity. Previous studies on critical period for visual cortex plasticity showed that dark rearing of mice from birth leads to prolonged visual critical period plasticity beyond juvenile period (Hensch, 2004). It would be interesting for future studies to examine to what extent pre-weaning (e.g., maternal deprivation) or post-weaning social experience (e.g., juvenile social isolation) impacts the timecourse of the sensitive period for social dominance hierarchy plasticity. Finally, our results demonstrate hierarchy stability modulation through plasticity regulators in male mice, however, female mice also form dominance hierarchies. Previous studies have shown female hierarchies are less dependent on experience (van den Berg et al., 2015), however, few studies have examined sex differences in neural mechanisms of dominance, social status, or the relative stability vs. plasticity of female hierarchies.

Overall, our results demonstrate that PFC-dependent social behaviors such as mouse social dominance hierarchies are regulated by mechanisms that modulate critical period plasticity in primary sensory cortical areas. These findings provide insight into mechanisms through which increased behavioral plasticity may be achieved to improve therapeutic recovery from psychiatric disorders that are associated with social deficits.

## Data Availability Statement

The raw data supporting the conclusions of this article will be made available by the authors, without undue reservation.

## Ethics Statement

The animal study was reviewed and approved by the Icahn School of Medicine at Mount Sinai Ethical Committee for animal research.

## Author Contributions

LB and HM designed and analyzed experiments and wrote the manuscript with inputs from all authors. LB, MP, and AT performed all behavior experiments. LB and AT performed immunohistochemistry. SA and HM supervised LB. All authors contributed to the article and approved the submitted version.

## Conflict of Interest

The authors declare that the research was conducted in the absence of any commercial or financial relationships that could be construed as a potential conflict of interest.

## References

[B1] Bavelier D. Levi D. M. Li R. W. Dan Y. Hensch T. K. (2010). Removing brakes on adult brain plasticity: from molecular to behavioral interventions. *J. Neurosci.* 30 14964–14971. 10.1523/jneurosci.4812-10.2010 21068299 PMC2992973

[B2] Béla C. Mónika B. Márton T. István K. (2007). Valproate selectively reduces EEG activity in anterior parts of the cortex in patients with idiopathic generalized epilepsy: a low resolution electromagnetic tomography (LORETA) study. *Epilepsy Res.* 75 186–191. 10.1016/j.eplepsyres.2007.06.009 17624734

[B3] Bicks L. K. Koike H. Akbarian S. Morishita H. (2015). Prefrontal cortex and social cognition in mouse and man. *Front. Psychol.* 6:1805. 10.3389/fpsyg.2015.01805 26635701 PMC4659895

[B4] Bicks L. K. Yamamuro K. Flanigan M. E. Kim J. M. Kato D. Lucas E. K. (2020). Prefrontal parvalbumin interneurons require juvenile social experience to establish adult social behavior. *Nat. Commun.* 11:1003. 32081848 10.1038/s41467-020-14740-z PMC7035248

[B5] Bukhari N. Burman P. N. Hussein A. Demars M. P. Sadahiro M. Brady D. M. (2015). Unmasking proteolytic activity for adult visual cortex plasticity by the removal of Lynx1. *J. Neurosci.* 35 12693–12702. 10.1523/jneurosci.4315-14.2015 26377459 PMC4571604

[B6] Chiu C.-T. Wang Z. Hunsberger J. G. Chuang D.-M. (2013). Therapeutic potential of mood stabilizers lithium and valproic acid: beyond bipolar disorder. *Pharmacol. Rev.* 65 105–142. 10.1124/pr.111.005512 23300133 PMC3565922

[B7] David H. A. (1987). Ranking from unbalanced paired-comparison data. *Biometrika* 74 432–436. 10.1093/biomet/74.2.432

[B8] Disney A. A. Aoki C. Hawken M. J. (2007). Gain modulation by nicotine in macaque v1. *Neuron* 56 701–713. 10.1016/j.neuron.2007.09.034 18031686 PMC2875676

[B9] Gammell M. P. De Vries H. Jennings D. J. Carlin C. M. Hayden T. J. (2003). David’s score: a more appropriate dominance ranking method than Clutton-Brock et al.’s index. *Anim. Behav.* 66 601–605. 10.1006/anbe.2003.2226

[B10] Gervain J. Vines B. W. Chen L. M. Seo R. J. Hensch T. K. Werker J. F. (2013). Valproate reopens critical-period learning of absolute pitch. *Front. Syst. Neurosci.* 7:102–102. 10.3389/fnsys.2013.00102 24348349 PMC3848041

[B11] Gordon J. A. Stryker M. P. (1996). Experience-dependent plasticity of binocular responses in the primary visual cortex of the mouse. *J. Neurosci.* 16 3274–3286. 10.1523/jneurosci.16-10-03274.1996 8627365 PMC6579137

[B12] Hensch T. K. (2004). Critical period regulation. *Annu. Rev. Neurosci.* 27 549–579. 10.1146/annurev.neuro.27.070203.144327 15217343

[B13] Ibañez-Tallon I. Miwa J. M. Wang H. L. Adams N. C. Crabtree G. W. Sine S. M. (2002). Novel modulation of neuronal nicotinic acetylcholine receptors by association with the endogenous prototoxin lynx1. *Neuron* 33 893–903. 10.1016/s0896-6273(02)00632-3 11906696

[B14] Kingsbury L. Huang S. Wang J. Gu K. Golshani P. Wu Y. E. (2019). Correlated neural activity and encoding of behavior across brains of socially interacting animals. *Cell* 178 429–446 e416.. 31230711 10.1016/j.cell.2019.05.022 PMC6625832

[B15] Lindzey G. Winston H. Manosevitz M. (1961). Social dominance in inbred mouse strains. *Nature* 191 474–476. 10.1038/191474a0 13762409

[B16] Machado-Vieira R. Ibrahim L. Zarate C. A. Jr. (2011). Histone deacetylases and mood disorders: epigenetic programming in gene-environment interactions. *CNS Neurosci. Ther.* 17 699–704. 10.1111/j.1755-5949.2010.00203.x 20961400 PMC3026916

[B17] Makinodan M. Rosen K. M. Ito S. Corfas G. (2012). A critical period for social experience-dependent oligodendrocyte maturation and myelination. *Science* 337 1357–1360. 10.1126/science.1220845 22984073 PMC4165613

[B18] Maya Vetencourt J. F. Sale A. Viegi A. Baroncelli L. De Pasquale R. O’leary O. F. (2008). The antidepressant fluoxetine restores plasticity in the adult visual cortex. *Science* 320 385–388. 10.1126/science.1150516 18420937

[B19] McGee A. W. Yang Y. Fischer Q. S. Daw N. W. Strittmatter S. M. (2005). Experience-driven plasticity of visual cortex limited by myelin and Nogo receptor. *Science* 309 2222–2226. 10.1126/science.1114362 16195464 PMC2856689

[B20] Miwa J. M. Ibanez-Tallon I. Crabtree G. W. Sanchez R. Sali A. Role L. W. (1999). lynx1, an endogenous toxin-like modulator of nicotinic acetylcholine receptors in the mammalian CNS. *Neuron* 23 105–114. 10.1016/s0896-6273(00)80757-6 10402197

[B21] Miwa J. M. Stevens T. R. King S. L. Caldarone B. J. Ibanez-Tallon I. Xiao C. (2006). The prototoxin lynx1 acts on nicotinic acetylcholine receptors to balance neuronal activity and survival in vivo. *Neuron* 51 587–600. 10.1016/j.neuron.2006.07.025 16950157

[B22] Morishita H. (2020). A prefrontal social circuit vulnerable to juvenile social isolation. *Neuropsychopharmacology* 46 229–230. 10.1038/s41386-020-00821-6 32873902 PMC7688936

[B23] Morishita H. Hensch T. K. (2008). Critical period revisited: impact on vision. *Curr. Opin. Neurobiol.* 18 101–107. 10.1016/j.conb.2008.05.009 18534841

[B24] Morishita H. Miwa J. M. Heintz N. Hensch T. K. (2010). Lynx1, a cholinergic brake, limits plasticity in adult visual cortex. *Science* 330 1238–1240. 10.1126/science.1195320 21071629 PMC3387538

[B25] Nelson A. C. Kapoor V. Vaughn E. Gnanasegaram J. A. Rubinstein N. D. Murthy V. N. (2019). Molecular and circuit architecture of social hierarchy. *bioRxiv* [preprint] bioRxiv:838664 doi: 10.1016/j.cell.2025.07.024 PMC12458795 40795854

[B26] Phiel C. J. Zhang F. Huang E. Y. Guenther M. G. Lazar M. A. Klein P. S. (2001). Histone deacetylase is a direct target of valproic acid, a potent anticonvulsant, mood stabilizer, and teratogen. *J. Biol. Chem.* 276 36734–36741. 10.1074/jbc.m101287200 11473107

[B27] Pizzorusso T. Medini P. Berardi N. Chierzi S. Fawcett J. W. Maffei L. (2002). Reactivation of ocular dominance plasticity in the adult visual cortex. *Science* 298 1248–1251. 10.1126/science.1072699 12424383

[B28] Putignano E. Lonetti G. Cancedda L. Ratto G. Costa M. Maffei L. (2007). Developmental downregulation of histone posttranslational modifications regulates visual cortical plasticity. *Neuron* 53 747–759. 10.1016/j.neuron.2007.02.007 17329213

[B29] Sajo M. Ellis-Davies G. Morishita H. (2016). Lynx1 limits dendritic spine turnover in the adult visual cortex. *J. Neurosci.* 36 9472–9478. 10.1523/jneurosci.0580-16.2016 27605620 PMC5013192

[B30] Silingardi D. Scali M. Belluomini G. Pizzorusso T. (2010). Epigenetic treatments of adult rats promote recovery from visual acuity deficits induced by long-term monocular deprivation. *Eur. J. Neurosci.* 31 2185–2192. 10.1111/j.1460-9568.2010.07261.x 20550570

[B31] Syken J. Grandpre T. Kanold P. O. Shatz C. J. (2006). PirB restricts ocular-dominance plasticity in visual cortex. *Science* 313 1795–1800. 10.1126/science.1128232 16917027

[B32] Takesian A. E. Bogart L. J. Lichtman J. W. Hensch T. K. (2018). Inhibitory circuit gating of auditory critical-period plasticity. *Nat. Neurosci.* 21 218–227. 10.1038/s41593-017-0064-2 29358666 PMC5978727

[B33] Timmer M. Sandi C. (2010). A role for glucocorticoids in the long-term establishment of a social hierarchy. *Psychoneuroendocrinology* 35 1543–1552. 10.1016/j.psyneuen.2010.05.011 20576360

[B34] van den Berg W. E. Lamballais S. Kushner S. A. (2015). Sex-Specific mechanism of social hierarchy in mice. *Neuropsychopharmacology* 40 1364–1372. 10.1038/npp.2014.319 25469681 PMC4397394

[B35] Wang F. Kessels H. W. Hu H. (2014). The mouse that roared: neural mechanisms of social hierarchy. *Trends Neurosci.* 37 674–682. 10.1016/j.tins.2014.07.005 25160682

[B36] Wang F. Zhu J. Zhu H. Zhang Q. Lin Z. Hu H. (2011). Bidirectional control of social hierarchy by synaptic efficacy in medial prefrontal cortex. *Science* 334 693–697. 10.1126/science.1209951 21960531

[B37] Williamson C. M. Klein I. S. Lee W. Curley J. P. (2019). Immediate early gene activation throughout the brain is associated with dynamic changes in social context. *Soc. Neurosci.* 14 253–265. 10.1080/17470919.2018.1479303 29781376

[B38] Williamson C. M. Lee W. Curley J. P. (2016). Temporal dynamics of social hierarchy formation and maintenance in male mice. *Anim. Behav.* 115 259–272. 10.1016/j.anbehav.2016.03.004

[B39] Yamaguchi Y. Lee Y. A. Kato A. Goto Y. (2017a). The roles of dopamine D1 receptor on the social hierarchy of rodents and nonhuman primates. *Int. J. Neuropsychopharmacol.* 20 324–335. 27927739 10.1093/ijnp/pyw106 PMC5409125

[B40] Yamaguchi Y. Lee Y. A. Kato A. Jas E. Goto Y. (2017b). The roles of dopamine D2 receptor in the social hierarchy of rodents and primates. *Sci. Rep.* 7:43348. 28233850 10.1038/srep43348 PMC5324123

[B41] Yamamuro K. Bicks L. K. Leventhal M. B. Kato D. Im S. Flanigan M. E. (2020). A prefrontal-paraventricular thalamus circuit requires juvenile social experience to regulate adult sociability in mice. *Nat. Neurosci.* 23 1240–1252. 10.1038/s41593-020-0695-6 32868932 PMC7898783

[B42] Yang E. J. Lin E. W. Hensch T. K. (2012). Critical period for acoustic preference in mice. *Proc. Natl. Acad. Sci. U.S.A.* 109(Suppl. 2) 17213–17220. 10.1073/pnas.1200705109 23045690 PMC3477391

[B43] Zhou T. Sandi C. Hu H. (2018). Advances in understanding neural mechanisms of social dominance. *Curr. Opin. Neurobiol.* 49 99–107. 10.1016/j.conb.2018.01.006 29428628

[B44] Zhou T. Zhu H. Fan Z. Wang F. Chen Y. Liang H. (2017). History of winning remodels thalamo-PFC circuit to reinforce social dominance. *Science* 357 162–168. 10.1126/science.aak9726 28706064

